# Supplementary material for: Crystalline Hydrate Dehydration Sensing Based on Integrated Terahertz Whispering Gallery Mode Resonators
Source: Sensors (Basel). 2022 Nov 24;22(23):9116. doi: 10.3390/s22239116 (PMC9736564; doi:10.3390/s22239116)
Supplement: Supplementary file 1 [file sensors-22-09116-s001.zip › sensors-1978494-supplementary.pdf]

Supporting Information

Crystalline hydrate dehydration sensing based on integrated terahertz whispering gallery mode resonators

Zhibo Hou [a], Shixing Yuan [a], Wentao Deng [a], Jiahua Cai [b], Yaqin Qiu [a], Yunong Zhao [a], Ziwei Wang [a], Liao Chen [a,*], Huan Liu [a,c,*], Xiaojun Wu [a,b,*], and Xinliang Zhang [a,c]

[a] Wuhan National Laboratory for Optoelectronics and School of Optical and Electronic Information, Huazhong University of Science and Technology, Wuhan 430074, China

[b] School of Electronic and Information Engineering, Beihang University, Beijing 100191, China

[c] Optics Valley Laboratory, Wuhan 430074, China

*Correspondence author: liaochenchina@hust.edu.cn

*Correspondence author: huan@mail.hust.edu.cn

*Correspondence author: xiaojunwu@buaa.edu.cn

Supplementary Note 1: The introduction of the operation process.

We note that the liquid-transfer method is a more accurate way of the quantitative transferring of tiny substances. The method is as follows: firstly, we prepare supersaturated $CuSO_4$ solution with a concentration of 2 mol/L at 80℃. Then as shown in Fig. S1(a), the $CuSO_4$ solutions are transferred on the surface of the sensor quantitatively and the minimum transferable volume of the pipette in the experiment is 0.1 μL. In experiment 3.1, firstly, one drop of 0.5 μL supersaturated $CuSO_4$ solutions is placed above the ring resonator. Then, we cool the supersaturated $CuSO_4$ liquid at 25℃ to crystallize. After cooling and crystallizing, one dot of 1 μmol $CuSO_4 \cdot 5H_2O$ is obtained above the ring resonator successfully.

In experiment 3.2, we follow the same method to transfer six drops of supersaturated $CuSO_4$ solutions with a concentration of 2 mol/L and a volume of 0.1 μL per drop on the surface of the THz-WGMR, and then we cool the liquids at 25 °C to obtain six dots of 0.2 μmol per dot $CuSO_4 \cdot 5H_2O$ as shown in Fig. S1(b). The numbers in Fig. S1(b) represent the dropping order, the number one is added to the sensor first, therefore, the number one crystallizes first.

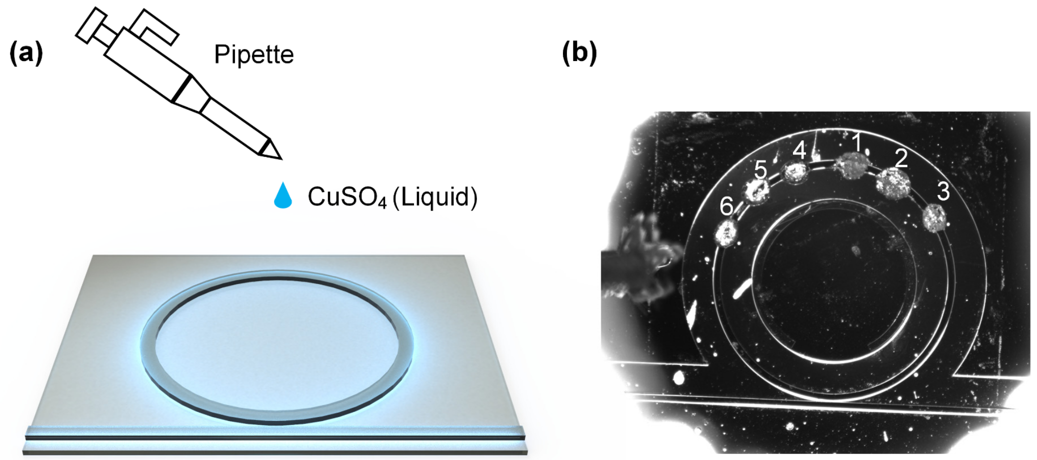

Figure S1. (a) The operation diagram. One drop of the supersaturated $CuSO_4$ solution of 0.5 μL (0.1 μL) is transferred above the ring resonator by the pipette. (b) Six drops of the $CuSO_4$ solution with a volume of 0.1 μL per drop covering the ring and the numbers represent the dropping order.

Supplementary Note 2: The illustration of the heating and maintaining equipment.

As shown in Fig. S2, the THz-WGMR is heated by a temperature-controlled heating plate above the sensor. The HRFZ-Si has a high thermal conductivity which can transfer heat to the crystal effectively. When the heating temperature is higher than 50 ℃, the $CuSO_4 \cdot 5H_2O$ gradually dehydrates and transforms into $CuSO_4 \cdot 3H_2O$; When the temperature is higher than 90 ℃, the $CuSO_4 \cdot 3H_2O$ gradually dehydrates and transforms into $CuSO_4 \cdot H_2O$ [1,2]. We adjust the temperature to change the water content with the benefit of keeping the amount and distribution of $CuSO_4 \cdot xH_2O$ (x=5, 3, 1) stable during the measurement. The heating equipment consists of an alumina heating plate together with an industrial temperature controller. The temperature accuracy of this controller is 1 ℃, and the maximum heating temperature reaches 150 ℃. The above heating equipment is used to fulfill the dehydration and transformation of the crystal in the experiment mainly. However, the temperature is related to the ER and resonance frequency of the resonance dip, which results from the thermo-optic effect [3]. In detail, the change in the temperature will change the absorption coefficient and the refractive index of the silicon, then lend to the change of the roundtrip length and the single-pass transmission coefficient of the ring.

To figure out the influence of the temperature on the ER and the resonant frequency of the resonant dip, we simulate and the results show that the temperature mainly affects the resonance frequency and the change of the resonance frequency with the temperature is ~20 MHz/℃ around 410 GHz [4,5]. Therefore, to ensure the stability and accuracy of the experimental results, a temperature control platform with a temperature control accuracy of 0.01℃ and a temperature variation range of 0~75 ℃ is placed under the chip to keep the temperature stable during the measurement. To ensure simplicity and clarity, only the heating platform under the chip is illustrated in Fig. 1(b), and the heating plate above the chip is not exhibited.

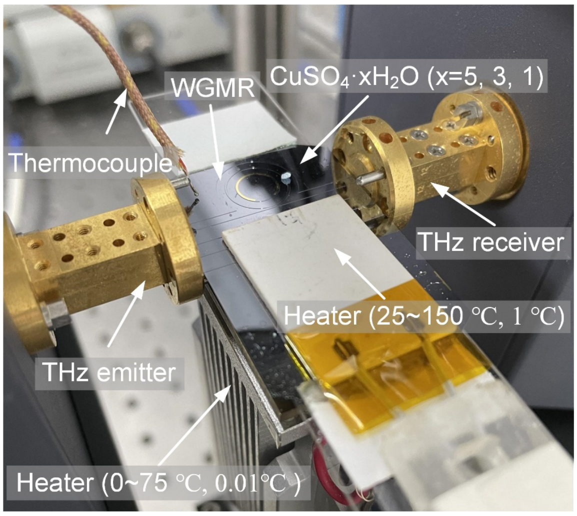

Figure S2. The diagram of the equipment.

Supplementary Note 3: The characterization of the hydration process.

The hydration of the $CuSO_4 \cdot H_2O$ is recorded and displayed in Fig. S3. Firstly, the $CuSO_4 \cdot H_2O$ with a weight of 1.389 g is placed on the electronic balance, then the changes of the weight are recorded to analyze the degree of the hydration. According to the molecular weight, the weight of the $CuSO_4 \cdot 3H_2O$ is 1.670 g after fully hydrating from the $CuSO_4 \cdot H_2O$. As implied in Fig. S3, during the recording time of 40 mins, the increment of the weight is 0.012 g, which means the hydration degree is less than five percent of the full hydration. As for the decrease in the weight at the beginning, there are two reasons, for the one hand, the uncertainty of the electronic balance is ±0.001 g. For another hand, the $CuSO_4 \cdot H_2O$ used in the experiment is from the dehydration of the $CuSO_4 \cdot 5H_2O$, we speculate that some residues still experience dehydration.

Noteworthy, the time of the temperature adjustment and stabilization of the temperature control platform is given by the operation manual.

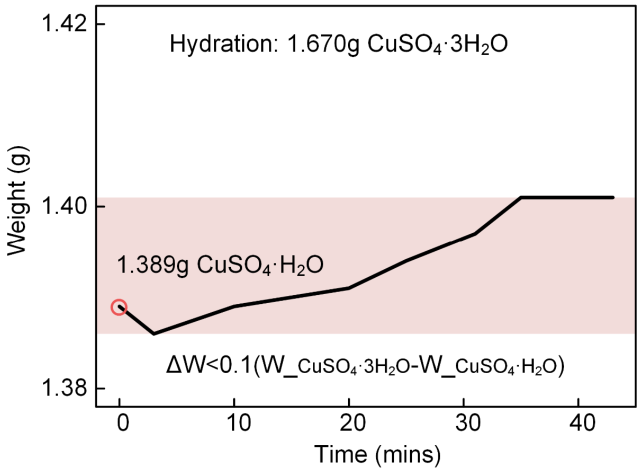

Figure S3. The weight curve of the $CuSO_4 \cdot H_2O$ during hydration.

Supplementary Note 4: The characterization of the operational deviation.

The variation of volume is 0.05 μL according to the standard deviation of the pipette. Furthermore, to evaluate the contribution of the variation of the volume to the ER and frequency shift, the variation of the volume should be transformed into the deviation of the overlapping areas between the drop and the ring. Therefore, the variation in the drop volume and the deviation of the overlapping areas are associated. As shown in Fig. S4, we measure the contact angles and contact lengths of six drops of the $CuSO_4$ solution with a volume of 0.1 μL per drop on the surface of the ring resonator. As implied in Table S1, the statistical contact angle and contact length are 63.7±2.2° and 1112.5±72.1 μm respectively. Due to the linear relationship between the frequency shifts and the contact length, it is convenient to estimate the operational deviation by the frequency shifts based on the deviation of the contact length (±6.5 %). The speculative deviations of the frequency shifts for one dot of 0.2 μmol $CuSO_4 \cdot xH_2O$ (x=5, 3, 1) are ±17.1 MHz, ±10.0 MHz, and ±7.3 MHz, respectively.

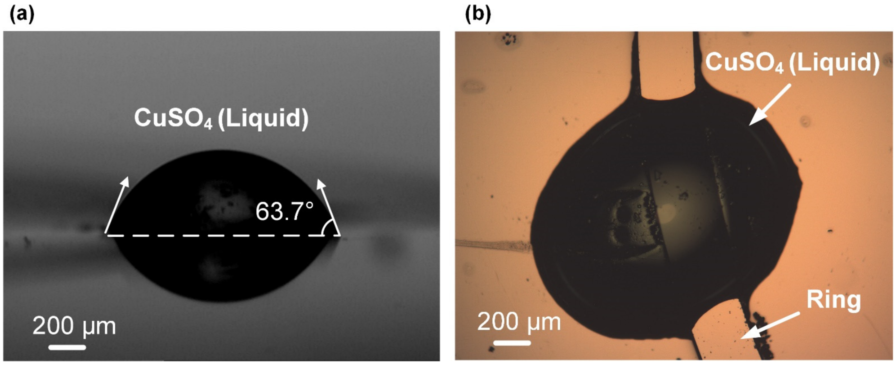

Figure S4. The optical microscopic images show the contact angle and contact length of the liquid drop. The side view (a) and the top view (b) of the 0.1 μL $CuSO_4$ drop.

Table S1. The contact angles and lengths of six drops of $CuSO_4$ solution

	Sample						Mean	Standard Deviation
	1	2	3	4	5	6		
Contact Angle (°)	62.46	64.85	63.24	67.56	61.80	62.25	63.69	2.18 (3.4 %)
Contact Length (μm)	1157	1037	1009	1156	1186	1130	1112.5	72.1 (6.5 %)

Supplementary Note 5: The description of the simulated process.

The simulation model is shown in Fig. S5. The $CuSO_4{\cdot}xH_2O$ (x=5, 3, 1) covers the ring resonator and the thickness of the sample is 200 μm shown as H_3. The H_1 and the H_2 are 60 μm respectively. We acquire the complex refractive indexes of the $CuSO_4{\cdot}xH_2O$ (x=5, 3, 1) using the THz-TDS. The setup is shown in Fig. S6. The results are shown in Fig. 4(a). We use the measured complex refractive indexes of $CuSO_4{\cdot}xH_2O$ (x=5, 3, 1) and the simulated model to calculate the complex mode refractive indexes, and the results are shown in Table S2. Besides, we use the experiment results in Fig. 3 measured by the THz-WGMR and the analytical model in Formula (2) to calculate the complex mode refractive indexes too. The corresponding results are shown in Table S2. We define that the relative errors (RR) of these two methods are calculated as

$$RR = \frac{V_{\text{THz-TDS}} - V_{\text{THz-WGMR}}}{V_{\text{THz-WGMR}}} \qquad \text{(S1.1)}$$

The RR of the mode refractive indexes caused by the $CuSO_4{\cdot}xH_2O$ (x=5, 3, 1) are 0.021, 0.003, and 0.011, and the RR of the mode extinction coefficients caused by the $CuSO_4{\cdot}xH_2O$ (x=5, 3, 1) are 0.19, 0.31, and 0.28, respectively as shown in Table S2.

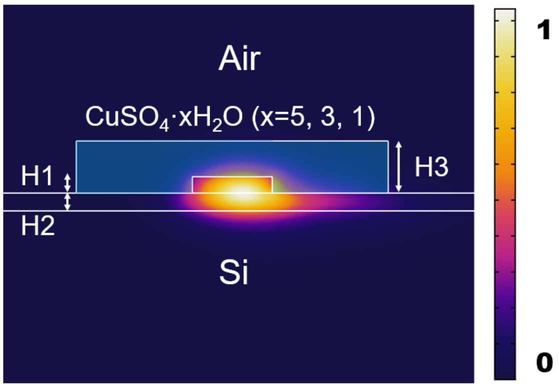

Figure S5. The simulated electric field distribution of the ridge waveguide of the ring resonator.

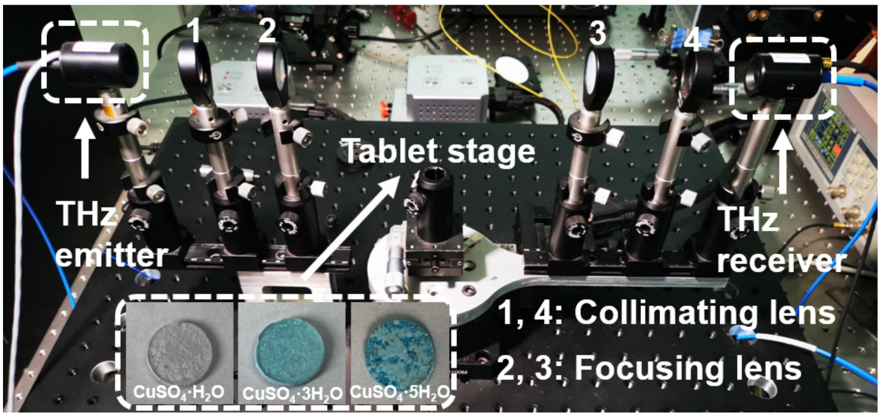

Figure S6. The setup of the THz-TDS consists of a THz emitter, a THz receiver, two collimating lenses, and two focus lenses. The tablet of the $CuSO_4 \cdot xH_2O$ (x=5, 3, 1) is loaded at the tablet stage.

Table S2. The mode refractive indexes and the mode extinction coefficients acquired by THz-TDS and THz-WGMR respectively.

Method		$CuSO_4 \cdot 5H_2O$	$CuSO_4 \cdot 3H_2O$	$CuSO_4 \cdot H_2O$
THz-TDS	mode refractive index	2.917	2.897	2.881
	mode extinction coefficient	0.0084	0.0054	0.0041
THz-WGMR	mode refractive index	3.040	2.945	2.907
	mode extinction coefficient	0.0105	0.0042	0.0033

References

1. White, R.L. Variable temperature infrared study of copper sulfate pentahydrate dehydration. *Thermochim. Acta* **2012**, *528*, 58-62, doi:https://doi.org/10.1016/j.tca.2011.11.013.
2. Fu, X.; Yang, G.; Sun, J.; Zhou, J. Vibrational Spectra of Copper Sulfate Hydrates Investigated with Low-Temperature Raman Spectroscopy and Terahertz Time Domain Spectroscopy. *The Journal of Physical Chemistry A* **2012**, *116*, 7314-7318, doi:10.1021/jp302997h.
3. Liu, L.; Dong, J.; Gao, D.; Zheng, A.; Zhang, X. On-chip passive three-port circuit of all-optical ordered-route transmission. *Sci. Rep.* **2015**, *5*, 10190, doi:10.1038/srep10190.
4. Yuan, S.; Chen, L.; Wang, Z.; Wang, R.; Wu, X.; Zhang, X. Tunable high-quality Fano resonance in coupled terahertz whispering-gallery-mode resonators. *Appl. Phys. Lett.* **2019**, *115*, doi:10.1063/1.5129073.
5. Vogt, D.W.; Jones, A.H.; Leonhardt, R. Thermal tuning of silicon terahertz whispering-gallery mode resonators. *Appl. Phys. Lett.* **2018**, *113*, 011101, doi:10.1063/1.5036539.
